# Efficacy of combined non-invasive brain stimulation and robot-assisted gait training on lower extremity recovery post-stroke: a systematic review and meta-analysis of randomized controlled trials

**DOI:** 10.3389/fneur.2025.1500020

**Published:** 2025-03-07

**Authors:** Jiaoyun Wang, Huihuang Zhang, Jiani Ma, Lei Gu, Xiang Li

**Affiliations:** ^1^The Third Affiliated Hospital of Zhejiang Chinese Medical University, Rehabilitation Assessment and Treatment Center, Hangzhou, Zhejiang, China; ^2^Shanghai Children’s Medical Center, Shanghai Jiaotong University School of Medicine, Shanghai, China; ^3^Department of Rehabilitation, Ningbo Medical Center Lihuili Hospital, Ningbo, China; ^4^Xiang'an Hospital of Xiamen University, School of Medicine, Xiamen University, Xiamen, China

**Keywords:** non-invasive brain stimulation, robot, rehabilitation, stroke, gait

## Abstract

**Background:**

Lower extremity dysfunction post-stroke significantly impedes patient independence and quality of life. Non-invasive brain stimulation (NIBS) and robot-assisted gait training (RAGT) have individually shown promising outcomes in gait recovery. However, the synergistic efficacy of non-invasive brain stimulation combined with robot-assisted gait training remains uncertain. This systematic review and meta-analysis aim to evaluate the combined therapy’s effectiveness on gait improvement and related motor functions in stroke patients.

**Methods:**

Following PRISMA guidelines, a comprehensive search was conducted to identify randomized controlled trials (RCTs) published up to September 2024. The primary outcome was assessed using the 6-min walk test (6MWT), with secondary outcomes examining assessed using the Functional Ambulation Category (FAC); the Motion Index (MI) to analyze exercise intensity; the Modified Ashworth Scale (MAS) to assess spasticity; and spatiotemporal gait parameters (SPG).

**Results:**

Six randomized controlled trials involving 191 stroke patients were included. Meta-analysis revealed that combined non-invasive brain stimulation and robot-assisted gait training significantly improved the 6-min walk test scores (mean difference [MD] = 21.81, 95% CI = 0.03–43.59), though effects on strength, activity participation, spasticity, and coordination were non-significant.

**Conclusion:**

Non-invasive brain stimulation combined with robot-assisted gait training shows potential in enhancing gait function but provides limited additional benefits for other motor functions. This combined approach may serve as an effective rehabilitation strategy for post-stroke gait recovery, warranting further large-scale studies to refine intervention protocols.

**Systematic review registration:**

https://www.crd.york.ac.uk/PROSPERO/view/CRD42021283890.

## 1 Introduction

Stroke ranks among the primary causes of long-term neurological impairment and mortality worldwide (1, 2). In the world, the crude incidence rate of stroke was 11.3 cases/1,000 person-years (3). The prevalence of stroke may be underestimated due to the cardiovascular effects of COVID-19 sequelae (1, 4). Disability following a cerebrovascular accident or stroke has become increasingly prominent (5). Independent walking is a critical determinant of overall autonomy and quality of life. Gait is also one of the most reliable indicators of physical function in stroke patients (6). It affects not only general health but also an individual’s independence in daily life (7). Major causes of gait abnormalities include limb weakness or paralysis, impaired balance, and reduced visual acuity (8). Rehabilitation interventions are essential for recovering functional abilities in stroke patients.

Rehabilitation robots stimulate muscle activation, synergy, and neural plasticity through specific repetitive movements and coordinated exercises. Compared to conventional rehabilitation therapy, lower-limb rehabilitation robots provide more robust neural stimulation, promoting the recovery of lower-limb function (9). Currently, lower-limb rehabilitation robots address most of the gait rehabilitation needs of stroke patients by improving standing balance (with and without assistance) (9, 10), enhancing walking ability (11), accelerating walking speed (10, 12), increasing joint mobility (especially in the hip and knee), strengthening leg muscles, improving motor patterns, and correcting abnormal gait (13, 14). These robots can enhance neural plasticity mechanisms related to motor learning and functional recovery after brain injury, including sensorimotor plasticity, effective frontal–parietal connectivity (FPEC), and cross-callosal inhibition (11, 15).

Non-invasive brain stimulation (NIBS), a simple, effective, and measurable rehabilitation technology, has been widely used in scientific research and clinical practice in recent years. It primarily includes transcranial magnetic stimulation (TMS) (16), transcranial alternating current stimulation (tACS), and transcranial direct current stimulation (tDCS) (17). Different paradigms and parameters can be applied according to specific conditions to modulate cortical excitability and regulate neural network functions (18).

A previous systematic review and meta-analysis evaluated tDCS as an intervention for stroke patients (19). Robot-assisted gait training, combined with physiotherapy and body weight support, appears to be an effective intervention for gait recovery following stroke (20). However, whether non-invasive brain stimulation, when used in conjunction with robot-assisted therapy, improves motor activity or function in stroke patients remains debated (21, 22). The primary objective of this systematic review and meta-analysis is to determine whether combining robot therapy with non-invasive brain stimulation can improve lower-limb walking function in stroke patients beyond what robot-assisted training alone can achieve. The secondary objective is to explore the potential synergistic effects of NIBS on robotic training. Additionally, we assess the effectiveness of NIBS in combination with RAGT in enhancing strength, reducing spasticity, and improving functional independence.

## 2 Methods

This systematic review and meta-analysis adhered to the Preferred Reporting Items for Systematic Reviews and Meta-Analyses (PRISMA) guidelines (23) and was registered in PROSPERO (registration ID: CRD42021283890).

### 2.1 Search strategy

Two authors (JYW and JNM) conducted a comprehensive literature search up to September 2024 across the professional databases MEDLINE (via PubMed), EMBASE, and the Cochrane Central Register of Controlled Trials (CENTRAL). To identify studies that may have been missed in database searches, the reference lists of all relevant papers were also manually reviewed. The databases were searched using the following keywords: gait, walking, robot, exoskeleton, non-invasive brain stimulation, transcranial direct current stimulation, and transcranial magnetic stimulation. The full search strategy is detailed in the Supplementary Table 1.

### 2.2 Selection criteria

**Figure 1:**
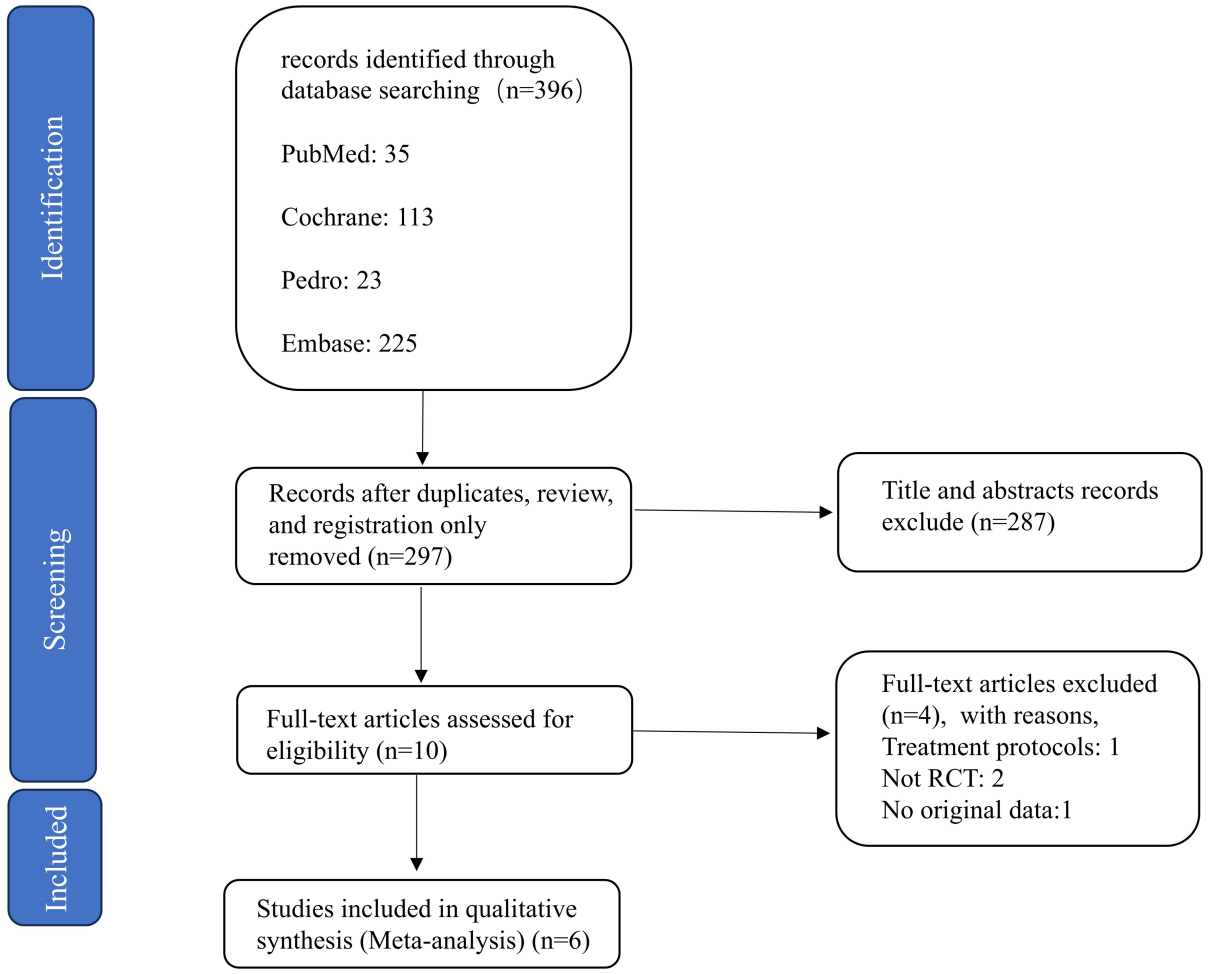
Flow of studies through the review.

The flowchart in Figure 1 illustrates the study selection process (24). Studies were selected according to the PICOS framework outlined in the PRISMA guidelines. We included studies based on the following criteria: (1) participants diagnosed with a cerebrovascular accident or stroke; (2) randomized controlled trials (RCTs); (3) combined intervention of non-invasive brain stimulation and robot-assisted walking training; (4) at least one assessment of lower limb function was conducted before and after the intervention.

Studies were excluded if they met the following criteria: (1) only protocols, abstracts, or conference papers; (2) participants younger than 18 years of age were included; (3) individuals with lower limb weakness from other conditions. Two independent investigators (JYW and JNM) selected the studies based on the inclusion and exclusion criteria. Any disagreements were resolved through discussion and reviewed by a third investigator (HHZ).

### 2.3 Data collection and extraction

Two researchers independently extracted data on the following parameters for the combination therapy and control groups: author, country, sample size, patient age and sex, type of intervention, randomization, time since stroke, inclusion and exclusion criteria, number of dropouts, side of cerebrovascular accident, type of stroke (ischemic/hemorrhagic), length and severity of rehabilitation, assessment period, and outcomes.

For outcome evaluation, the 6-min walking test (6MWT) was the primary measure of physical function, as it reflects cardiopulmonary function and exercise tolerance and is one of the best predictors of community walking (2, 25). Secondary measures included activity participation [assessed using the Functional Ambulation Category (FAC)]; the Motion Index (MI) to analyze exercise intensity; the Modified Ashworth Scale (MAS) to assess spasticity; and spatiotemporal gait parameters (SPG) described by step frequency and the ratio of single-phase to dual-phase support periods. We selected the ratio of single-phase to dual-phase support periods as the outcome to describe physical coordination and symmetry, as it provides a more comprehensive assessment than step frequency alone. Follow-up was assessed by recording the number of adverse events and dropouts in each group.

Three comparisons were made in 3-arm studies: according to the recommendations of the Cochrane Group, the shared group in the study was divided into smaller subgroups to ensure the independence of comparisons. In Picelli et al. (26), Group 2, which received sham tDCS combined with cathodal tsDCS (*n* = 10), was designated as the shared group. This group was compared with Group 1, which received anodal tDCS combined with sham tsDCS (*n* = 10), and Group 3, which received tDCS combined with cathodal tsDCS (*n* = 10). Consequently, the number of participants in Group 2 was divided by 2. In Geroin et al. (27), Group 1, which underwent RAGT combined with tDCS (*n* = 10), was defined as the shared group. This group was compared with Group 2, which underwent RAGT combined with sham tDCS (*n* = 10), and Group 3, which performed overground walking (*n* = 10). As a result, the number of participants in Group 1 was divided by 2. After confirming normal distribution, data collected as medians and quartiles were transformed into means and standard deviations (28, 29).

For studies requiring clarification or access to missing data, corresponding authors were contacted for further details.

### 2.4 Quality assessment

The quality of the six included studies was assessed using the Cochrane Collaboration’s Risk of Bias (RoB) 1.0 tool. Two reviewers (JNM and XL) assessed potential bias using the following criteria: (1) random sequence generation, (2) allocation concealment, (3) blinding of participants and personnel, (4) selective outcome assessment, (5) incomplete outcome data, (6) selective reporting, and (7) other bias. Disagreements were resolved through consensus. The analysis was performed using RevMan 5.4 (The Cochrane Collaboration, London, United Kingdom).

To assess publication bias, funnel plots for the primary variable (function) were examined. Duval and Tweedie’s trim-and-fill method was used to adjust effect estimates by accounting for unpublished studies with smaller effects.

### 2.5 Statistical analysis

The feasibility of a meta-analysis was assessed following data extraction. A meta-analysis was conducted if the intervention procedures and outcomes of at least three studies were comparable. The meta-analysis was performed using RevMan 5.4 software. When certain outcomes were presented on different scales, they were represented using the standardized mean difference (SMD). If all studies used the same measurement tool, mean difference (MD) was calculated. To account for adverse events and loss to follow-up, risk difference (RD) was computed. For each outcome, a 95% confidence interval (95% CI) was calculated.

Publication bias was assessed using a funnel plot. Heterogeneity was assessed using the *I*^2^ statistic. Statistical heterogeneity was considered significant when *I*^2^ ≥ 50% (30). Sensitivity analysis was performed to assess the potential contribution of high-risk studies to heterogeneity and their impact on result stability. Results were considered statistically significant when the *p*-value was less than 0.05 in the corresponding *z*-test.

## 3 Results

### 3.1 Included studies and main characteristics

In the final search conducted in January 2025, 396 records were identified from the databases. Ninety-nine duplicates, review, and registration only were identified, and after screening the titles and abstracts, 287 records were excluded. The full texts of 10 articles were examined for eligibility. Furthermore, 4 articles were excluded based on the following five exclusion criteria: (1) non-randomized studies (*n* = 2), (2) treatment protocols (*n* = 1), (3) no original data (*n* = 1). Finally, the systematic review and meta-analysis included six RCTs (26, 27, 31–34) that met the inclusion criteria (Figure 1).

The six studies included non-invasive brain stimulation and robot-assisted walking training, with a total of 191 participants (126 females, 66.0%; 98 participants in the intervention group, 51.3%). The maximum mean age was 75.25 years, and the minimum mean age was 52.7 years. In four studies (26, 32–34), the stroke onset time was more than 6 months; in one study (31) the onset time was between 2 weeks and 6 months (subacute stroke); and in one study (27), the onset time exceeded 12 months. Treatment frequency was five times per week. Treatment lasted for 2 weeks (26, 27, 32–34) or 4 weeks (31). The treatment duration ranged from 20 to 65 min. Except for the control group, there was no difference in treatment duration between the experimental and control groups. Only one of the six studies did not include follow-up (31). Table 1 displays the sociodemographic and clinical characteristics of the study participants. Table 2 lists the study’s intervention measures and evaluation methods.

**Table 1:** Characteristics of the participants of the studies included in the meta-analysis.

Study	Classification	Patients (n)	Age, years Mean (SD)	Gender M/F	Months poststroke, Mean (SD)	Type I/H	Affected hemisphere R/L	Location of lesion C/SC/MX
Picelli et al. (2019) (34)	Chronic (>6 month)	40	64.8 (10.1)	23/17	64.1 (44.2)	NR	NR	14/14/12
Picelli et al. (2018) (33)	Chronic (>6 month)	20	62.7 (9.9)	7/13	59.5 (43.6)	NR	NR	7/7/6
Seo et al. (2017) (32)	Chronic (>6 month)	21	62.0 (8.7)	16/5	112.2 (108.7)	16/5	13/8	NR
Leon et al. (2017) (31)	Subacute (>2 weeks and ≤6 months)	50	48.0 (11.0)	35/15	1.9 (1.1)	29/21	26/24	NR
Picelli et al. (2015) (26)	Chronic (>6 month)	30	62.3 (6.8)	22/8	56.0 (33.8)	NR	NR	11/8/11
Geroin et al. (2011) (27)	Chronic (>12 month)	30	62.7 (6.4)	23/7	26.4 (5.5)	NR	NR	12/9/9

Male (M); Female (F); Standard Deviation (SD); Ischaemic (I); Haemorrhagic (H); Right (R); Left (L); Cortical (C); Subcortical (SC); Mixed (MX).

**Table 2:** Interventions reported in the meta-analysis.

Study	Robotic device	NIBS	Therapy protocol	Follow-up	Outcome measure
Picelli et al. (2019) (34)	G-EO System Evolution	Cathodal tcDCS + Cathodal tsDCS	10 Sessions of 20 min of tcDCS and tsDCS while RAGT during 2 weeks.	2 weeks, 4 weeks	6MWT, FAC, MI, MAS, SGP
Picelli et al. (2018) (33)	G-EO System Evolution	Cathodal tcDCS + Cathodal tsDCS	10 Sessions of 20 min of tcDCS and tsDCS while RAGT during 2 weeks.	2 weeks, 4 weeks	6MWT, FAC, MI, MAS, SGP
Seo et al. (2017) (32)	Walkbot	Anodal tDCS	10 Sessions of 20 min of anodal tDCS, followed by 35 min RAGT during 2 weeks.	4 weeks	6MWT, FAC, 10MWT, BBS, FMA-LE, MRC, EMG activity, MEP
Leon et al. (2017) (31)	Lokomat	Anodal tDCS	20 Sessions of 20 min of tDCS, followed by 30–45 min RAGT during 4 weeks.	Immediately Post-intervention	10MWT, FAC
Picelli et al. (2015) (26)	G-EO System Evolution	Anodal tDCS + Cathodal tsDCS	10 Sessions of 20 min of tcDCS and tsDCS while RAGT during 2 weeks.	2 weeks, 4 weeks	6MWT, FAC, MI, MAS, SGP
Geroin et al. (2011) (27)	Gait Trainer GT1	Anodal tDCS	10 Sessions of 7 min of tDCS while 50 min RAGT during 2 weeks.	2 weeks	FAC, SGP, MI, MAS

Robot-assisted gait training (RAGT). Transcranial direct current stimulation (tDCS). Transcranial direct current stimulation of the cerebellum (tcDCS). Transcutaneous spinal direct current stimulation (tsDCS). Six minute walk test (6MWT). Ten minute walking test (10MWT). Spatiotemporal gait parameters (SGP). The Motricity Index leg sub-score (MI). Modified Ashworth scale (MAS). Fugl-Meyer assessment scale for lower extremities (FMA-LE). Medical Research Council sum score (MRC). Berg balance scale (BBS). Functional ambulation category (FAC). Electromyography (EMG). Motor Evoked Potentials (MEP).

### 3.2 Risk of bias in the included studies

**Figure 2:**
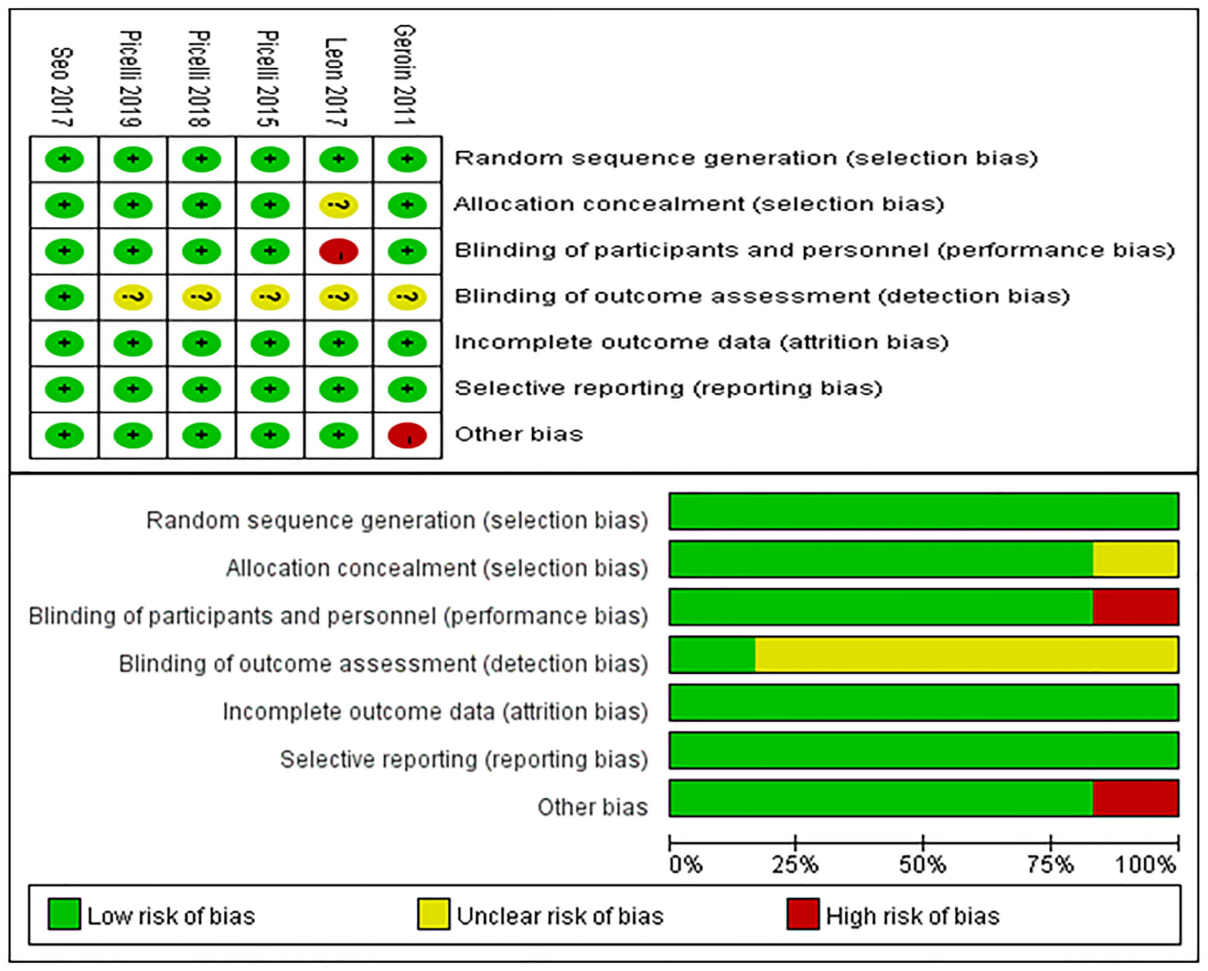
Risk of bias summary. Review authors’ judgments about each risk of bias item for each included study (upper figure). Risk of bias item presented as percentages across all included studies (lower figure).

Figure 2 illustrates the risk of bias in the included controlled studies. Risk of bias was assessed in the selected randomized controlled trials. One study (27) did not implement blinding due to the establishment of a blank control group, while three studies (31, 33, 34) used single-blind methods, and two studies (26, 32) used double-blind methods. For random sequence generation, one study (31) did not specify the method of sequence generation, while the other studies (26, 27, 32–34) demonstrated adequate randomization. Allocation concealment was implemented in four studies (26, 32–34), but was not reported in two studies (27, 31). Only one study (32) was considered to have blinded outcome assessment. All studies (26, 27, 31–34) were considered to have complete data. Only one study (27) was considered to have other biases due to its early publication date.

**Figure 3:**
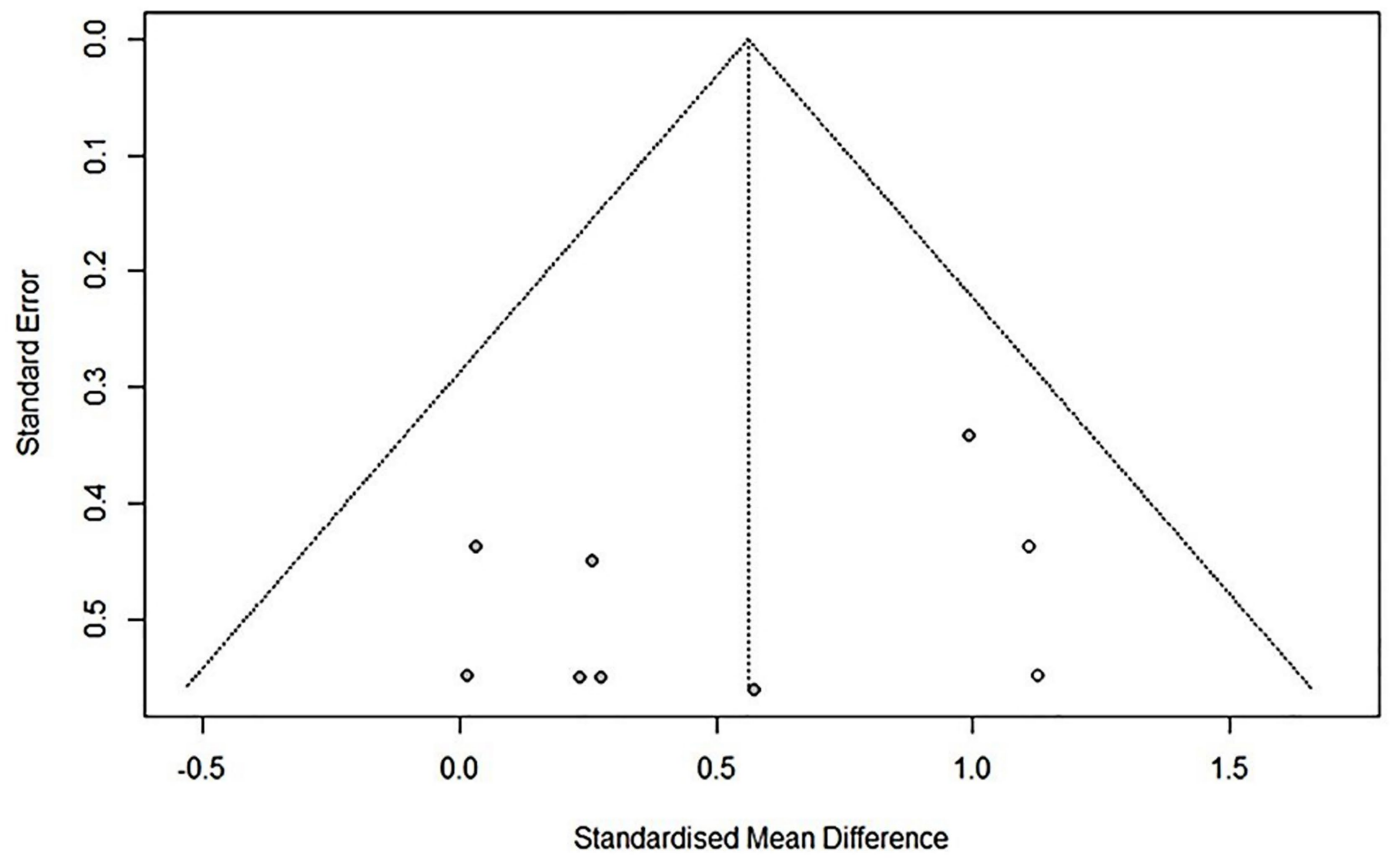
Funnel plot modified by trim and fill method comparison between RAGT associated with NIBS (active) versus RAGT associated with sham/none NIBS (control) for the main outcome. Asymmetries were not observed.

The trim-and-fill method was used to adjust for asymmetry in the funnel plot’s main variable distribution. The corrected results are presented in Figure 3. The distribution of variables in the funnel plot using the trim-and-fill method was symmetrical, indicating a low risk of publication bias.

### 3.3 Quantitative summary

A quantitative analysis of the primary outcome, lower limb function, was conducted following the objectives of this meta-analysis and the PROSPERO protocol. Lower limb function was assessed using the six-minute walk test (6MWT), which reflects cardiopulmonary function and exercise tolerance, and is a strong predictor of community walking (2, 25). The effect on strength was investigated in five studies (26, 27, 32–34). The effects on activities and participation were investigated in six studies (26, 27, 31–34) spasticity in three studies (26, 33, 34), and physical coordination was measured using the ratio of single- to double-support duration in four studies (26, 27, 33, 34).

**Figure 4:**
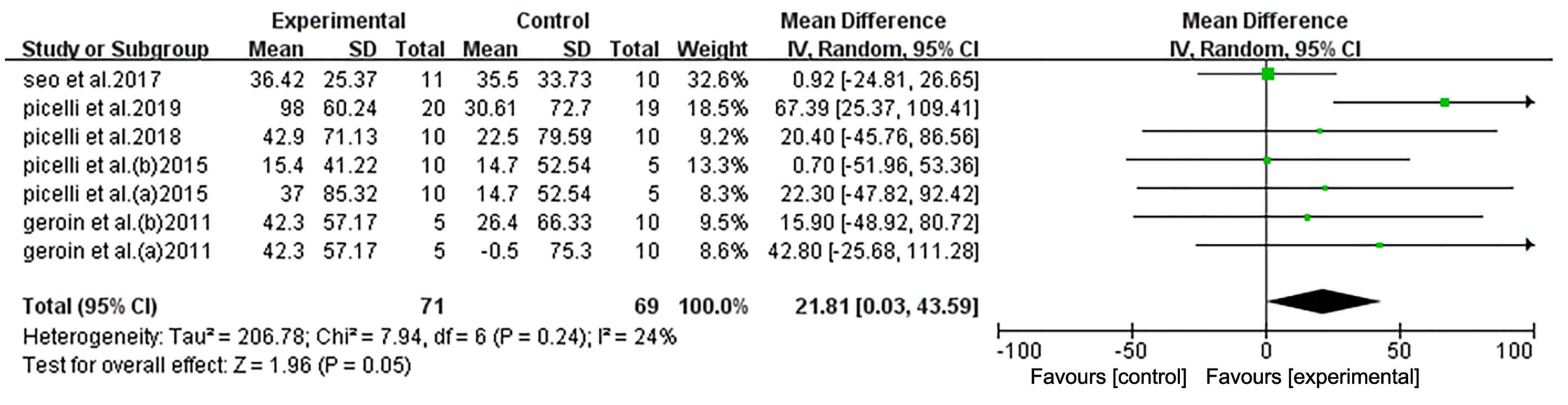
Forest plot of all trials comparing RAGT associated with NIBS (active) versus RAGT associated with sham/none NIBS (control) for body structure/function outcomes.

Among the RCTs, five studies performed the 6MWT and were divided into seven groups. Pooled data demonstrated that, compared to the control group, NIBS combined with RAGT significantly improved lower limb function (mean difference [MD] = 21.81, 95% confidence interval [CI] = 0.03–43.59, *n* = 140), with low heterogeneity (*I*^2^ = 24%, *p* = 0.24) (Figure 4).

**Figure 5:**
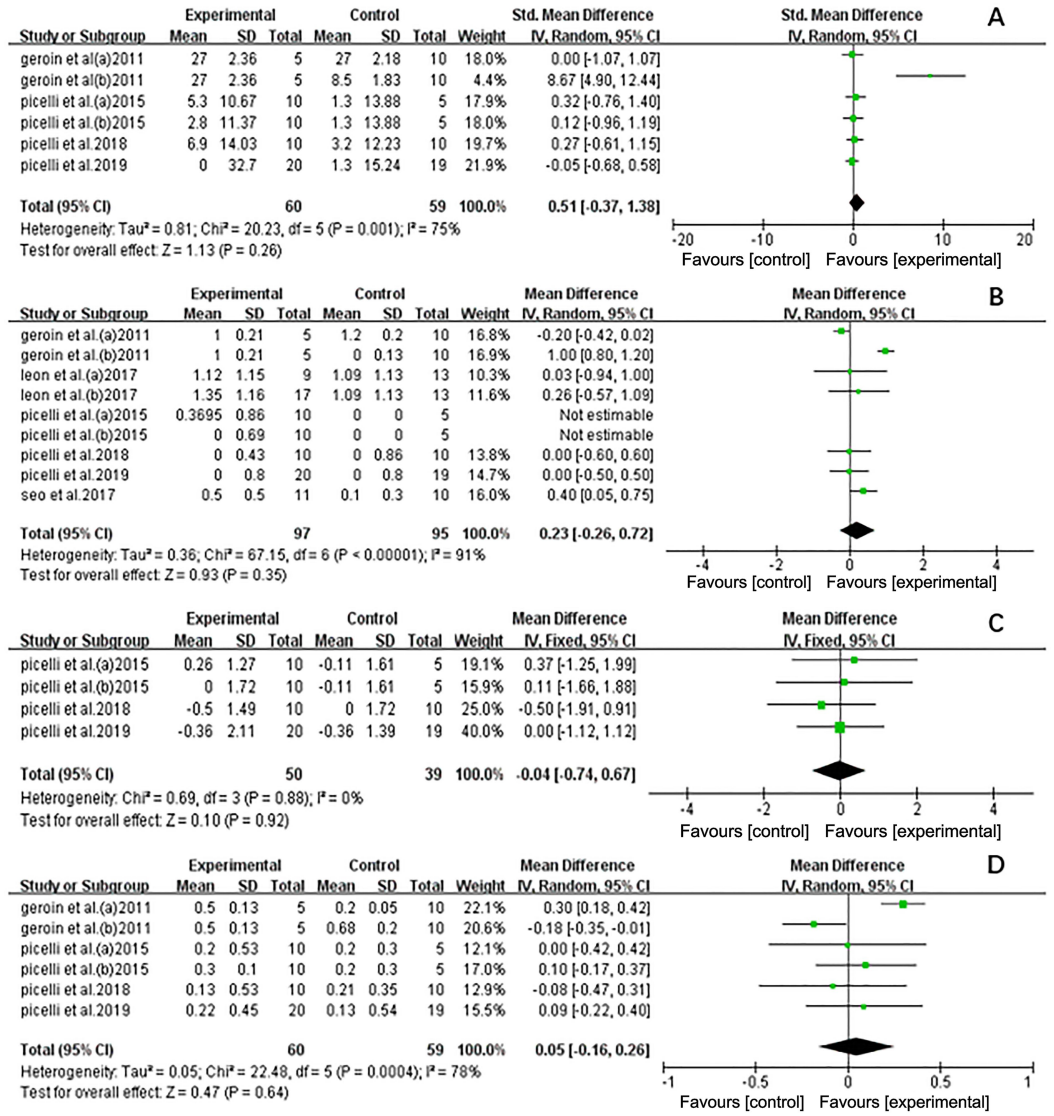
Forest plot of all trails comparing RAGT associated with NIBS (active) versus RAGT associated with Sham/none NIBS (control) for secondary outcomes. **(A)** Effect on strength. **(B)** Effect on activities and participation. **(C)** Effect on spasticity. **(D)** Effect on coordination.

The meta-analysis indicated that NIBS did not enhance the effects of RAGT in improving strength in stroke patients (standardized mean difference [SMD] = 0.51, 95% CI: −0.37 to 1.38, *n* = 119) and displayed considerable heterogeneity (*I*^2^ = 75%, *p* < 0.01) (Figure 5A). Geroin et al. (27) significantly influenced the results. When excluded (*I*^2^ = 0%; *p* = 0.97), a random-effects model was applied (SMD = 0.09; 95% CI: −0.30 to 0.49; *p* = 0.65), and heterogeneity decreased compared to the initial analysis (*I*^2^ = 75%, *p* < 0.01). The sensitivity analysis results remained stable, suggesting that the excluded study was a source of heterogeneity (Supplementary Figure 1).

The Functional Ambulation Category (FAC) scores were used to represent activities and participation. The analysis of NIBS combined with RAGT did not reveal any significant effects on activities and participation (MD = 0.23, 95% CI = −0.26 to 0.72, *n* = 192) and showed considerable heterogeneity (*I*^2^ = 91%, *p* < 0.01) (Figure 5B). Geroin et al. (27) also significantly influenced the results. When excluded (*I*^2^ = 0%; *p* = 0.66), a random-effects model was applied (MD = 0.21; 95% CI = −0.03 to 0.45; *p* = 0.08), and heterogeneity decreased (*I*^2^ = 91%, *p* < 0.01). The sensitivity analysis results remained stable, suggesting that the excluded study was a source of heterogeneity (Supplementary Figure 2).

Spasticity was assessed using the Modified Ashworth Scale (MAS). Figure 5C shows the pooled results. The results showed that NIBS-RAGT had no significant effect on spasticity compared with control interventions (MD = −0.04; 95% CI = −0.74 to 0.67, *n* = 89), and the data revealed low heterogeneity (*I*^2^ = 0%, *p* = 0.88).

The trials assessing the effect on physical coordination are summarized in Figure 5D. The total effect on physical coordination did not reveal a significant difference between the experimental and control groups (MD = 0.05; 95% CI = −0.16 to 0.26, *n* = 119), with high heterogeneity (*I*^2^ = 78%, *p* < 0.01). Similar findings were observed in a sensitivity analysis excluding studies with a high risk of bias (MD = −0.06; 95% CI = −0.18 to 0.07; *p* = 0.37), which reduced heterogeneity (*I*^2^ = 6%; *p* = 0.37) (Supplementary Figure 3).

### 3.4 Adverse effects

**Figure 6:**
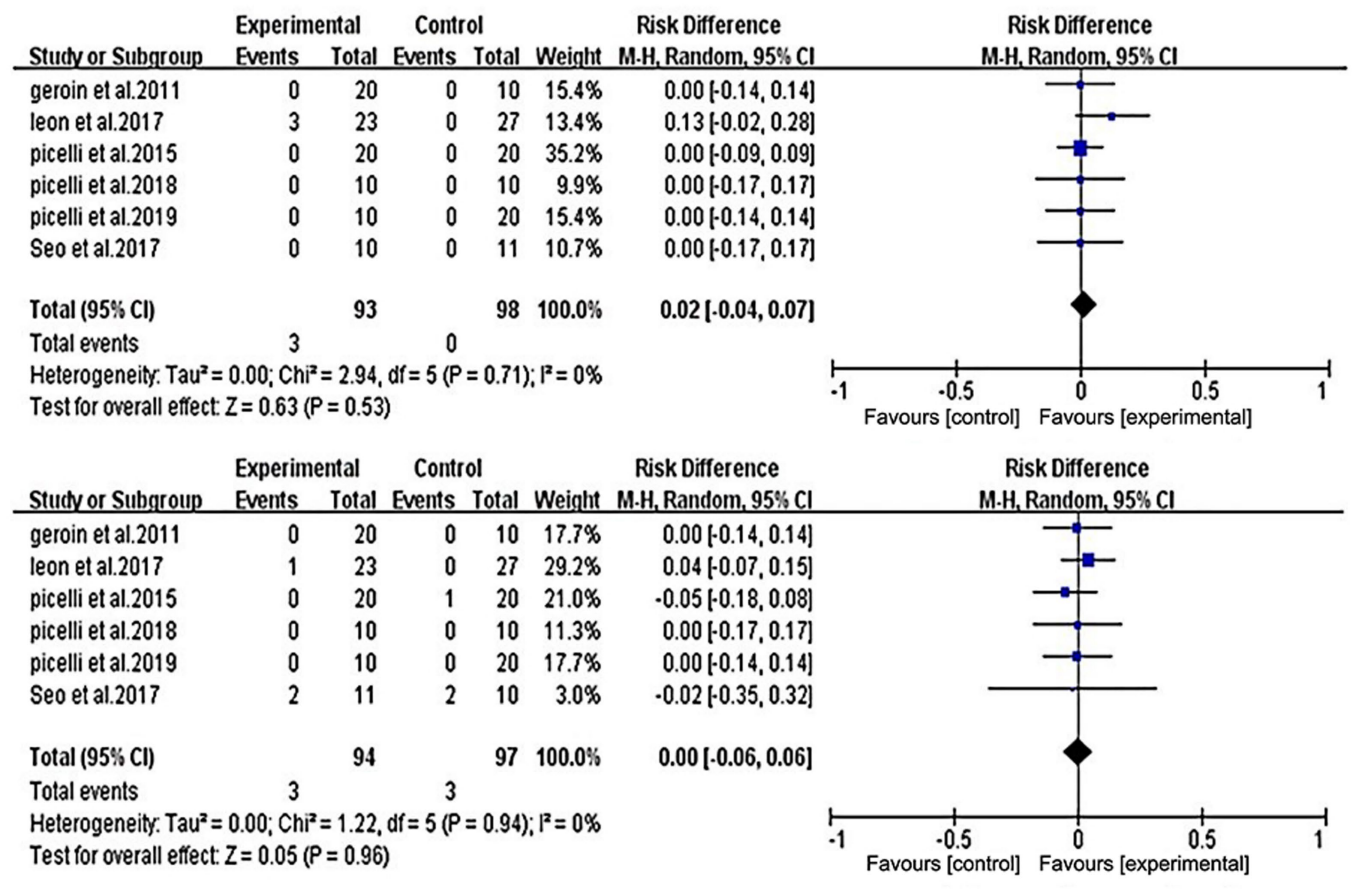
Forest plot of comparison between RAGT associated with NIBS (active) versus RT associated with sham/none NIBS (control) for adverse event (upper figure) and lost to follow-up (lower figure).

All studies were included in the analysis. Studies documenting adverse events and the number of patients lost to follow-up are listed in Figure 6. The analysis showed no significant difference between the two groups in the risk of adverse events (RD = −0.02; 95% CI = −0.04 to 0.07) or in the number of patients lost to follow-up (RD = 0.00; 95% CI = −0.06 to 0.06). All six included studies reported on adverse reactions; however, only Leon et al. (31) in the experimental group reported two cases of temporary mild pruritus and one case of exclusion due to mild headache during and after stimulation.

## 4 Discussion

This systematic review and meta-analysis summarize the clinical effects of NIBS-Robot training on lower extremity function, safety profiles, strength, spasticity, activities, participation, and physical coordination in stroke patients. The NIBS protocols in the study included transcranial direct current stimulation (tDCS), cerebellar transcranial direct current stimulation (tcDCS), and transcutaneous spinal direct current stimulation (tsDCS) spatiotemporal gait parameters. Currently, evidence on the effectiveness of NIBS-RAGT in previous clinical trials remains inconclusive. The meta-analysis indicated a substantial improvement in the primary outcome (lower limb function) and non-significant improvements in the secondary outcomes (strength, spasticity, activities, participation, and physical coordination).

Ambulation involves motor patterns generated by central pattern generators (CPGs) and cortical and subcortical structures (35). Due to the complexity of motion control, NIBS combined with robotic therapy may better support the functional reorganization of motor networks (36). The NIBS protocols in this review were divided into two strategies: direct stimulation of the affected M1 and indirect stimulation through the dentate-thalamo-cortical pathway, both based on the interhemispheric competition model. This hypothesis may limit the benefits for some stroke patients whose corticospinal tracts are severely damaged (18). Individualized NIBS therapy, tailored to patient conditions, may play a stronger role in enhancing robotic therapy (37). In this meta-analysis, heterogeneity was mainly attributed to Geroin et al. (27), likely due to the different types of robots and NIBS paradigms. Interestingly, the placebo effect of sham transcranial direct current stimulation was investigated, revealing insights into the mechanism of intervention (38). Additionally, factors such as NIBS parameters, electrode size, stimulation site, duration, and frequency may influence the intervention’s efficacy (39). Individual variability in NIBS includes differences in brain anatomy, cerebral cortex activity, muscle precontractions, focus of attention, and even variability caused by menstrual cycles and circadian rhythms (40). Non-invasive brain stimulation, particularly transcranial direct current stimulation, has shown potential for improving motor function in patients with neurological impairments. However, the results are often inconsistent, which can be attributed in part to the standardized, non-tailored approach used in many studies. A key limitation of current tDCS protocols is their reliance on fixed stimulation parameters, such as electrode placement, stimulation intensity, and polarity (anodal vs. cathodal). These protocols do not account for the individual variability in neurophysiological responses across patients. Future research could benefit from a more tailored approach, where tDCS is adjusted to preserve critical neural pathways, such as the corticospinal tract, and guided by motor evoked potentials (MEPs). This individualized approach, in which both stimulation parameters and electrode sites are customized to each patient’s unique brain activity, may enhance the efficacy of tDCS and lead to more consistent improvements in motor function (17). In the future, neuronavigation and real-time EEG monitoring of NIBS effects may help reduce variability in outcomes (41). Clarifying the response mechanisms and biomarkers may be key to improving success rates.

While the primary outcomes demonstrated significant improvement, the secondary outcomes did not show marked changes. A closer look at the potential reasons for this discrepancy reveals the influence of different robotic-assisted gait training (RAGT) systems and experimental designs. One such system is the Lokomat (42), a robotic gait orthosis designed to assist patients with impaired mobility by providing repetitive, adjustable movement patterns during walking. The Lokomat system is equipped with adjustable robotic legs that support the patient’s lower limbs while encouraging a natural gait pattern, offering a combination of body weight support and assistance in leg movement. The system can be calibrated for different levels of assistance, ranging from low to high intensity, based on individual patient needs. However, the impact of the Lokomat on secondary outcomes such as gait performance may be limited if the system is not tailored to the specific therapeutic goals of the patient (43). Furthermore, the protocols used in these interventions—including session duration, frequency, and intensity—might also explain the limited changes observed in secondary outcomes. These factors highlight the complexity of evaluating gait improvement and motor function in clinical settings, where secondary outcomes might not always align with the primary measures of success.

Regarding adverse events, NIBS-Robot training can be considered a safe treatment. All studies reported that NIBS-Robot training was safe, with no severe adverse events. Only one included study observed mild adverse reactions, consistent with earlier research (44).

Robots in this study were categorized as exoskeletons or end-effector devices. All exoskeleton robots in the included studies were static, utilizing a fixed treadmill. Compared to overground robots, the combination with NIBS may involve different mechanisms (45). Moreover, in all randomized controlled trials, the robot settings were not adequately tailored to patient characteristics. Larger and more comparative studies are needed to determine the optimal timing and program design for individualized treatment.

Limitations of this meta-analysis include: (1) we included only six studies for meta-analysis, with three from the same group (Picelli et al.). This limits the generalizability of our findings due to potential biases in study design and sample selection. Future research should incorporate more diverse data to improve result reliability. (2) Most subjects had chronic stroke, so findings cannot be extended to patients with subacute stroke. (3) The sample sizes in the included studies were generally small, with only one study reaching 50 participants (31). Larger sample sizes may influence the outcomes. (4) Differences between intervention programs and equipment. (5) A lack of neurophysiological assessments of cortical excitability and brain connectivity, with only one study evaluating motor evoked potentials (MEP) via transcranial magnetic stimulation. (6) The majority of trials lacked long-term follow-up. (7) All NIBS treatments followed the interhemispheric competition model, which may not be suitable for all stroke patients.

## 5 Conclusion

The meta-analysis showed that NIBS combined with RAGT may be beneficial for lower limb function, but these results should be interpreted cautiously due to the limitations of this study. Furthermore, no significant improvements were found for NIBS combined with RAGT in terms of strength, spasticity, activities, participation, or physical coordination. Regarding adverse events, NIBS combined with RAGT can be considered a safe treatment with few adverse reactions. Moreover, the heterogeneity of subjects, variability of NIBS settings and RAGT devices, and unpredictability of results make comparisons between trials difficult. Future RCTs need to stratify patients based on the type and stage of cerebrovascular accident, include larger sample sizes, longer follow-up periods, and consider adverse reactions to determine the optimal parameters of NIBS combined with RAGT.

## Data Availability

The original contributions presented in the study are included in the article/Supplementary material, further inquiries can be directed to the corresponding author.
