# Supplementary material for: Efficacy of combined non-invasive brain stimulation and robot-assisted gait training on lower extremity recovery post-stroke: a systematic review and meta-analysis of randomized controlled trials
Source: Front Neurol. 2025 Mar 7;16:1500020. doi: 10.3389/fneur.2025.1500020 (PMC11925768; doi:10.3389/fneur.2025.1500020)
Supplement: Supplementary file 2 [file Supplementary_file_2.docx]

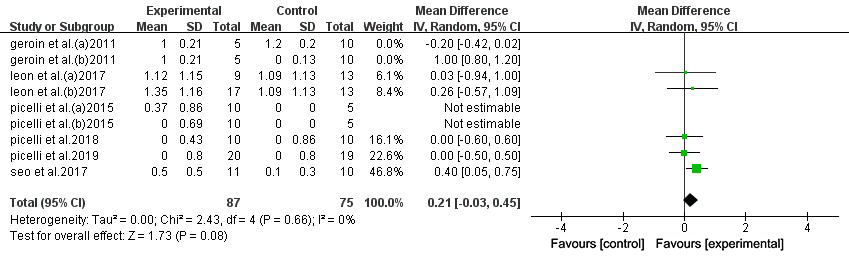


**Supplementary Figure 2.** Forest plot of sensitivity analysis by excluding studies with a high risk of bias. The pooled effect size measures FAC no improvement compared to control.
