# Supplementary material for: Efficacy of combined non-invasive brain stimulation and robot-assisted gait training on lower extremity recovery post-stroke: a systematic review and meta-analysis of randomized controlled trials
Source: Front Neurol. 2025 Mar 7;16:1500020. doi: 10.3389/fneur.2025.1500020 (PMC11925768; doi:10.3389/fneur.2025.1500020)
Supplement: Supplementary file 4 [file Table_1.docx]

**Appendix**

**Table.1 Search strategy**

| **Literature search (last search performed Jan 7, 2025)** | | **Number** |
| --- | --- | --- |
| PubMed | #1 stroke[MeSH Terms] OR 'cerebrovascular disorders' [MeSH Terms] OR 'brain infarction' [MeSH Terms] OR stroke[Title/Abstract] OR 'Cerebrovascular Accident' [Title/Abstract] OR Apoplexy[Title/Abstract]  #2 Locomotion[MeSH Terms] OR gait[Title/Abstract] OR walking[Title/Abstract] OR 'lower extremit*' [Title/Abstract] OR 'lower limb*' [Title/Abstract]  #3 robotics[MeSH Terms] OR 'orthotic devices' [MeSH Terms] OR 'body weight' [MeSH Terms] OR 'exoskeleton device' [MeSH Terms] OR robot[Title/Abstract] OR exoskeleton[Title/Abstract]  #4 'transcranial magnetic stimulation' [MeSH Terms] OR 'transcranial direct current stimulation' [MeSH Terms] OR 'transcranial magnetic stimulation' [Title/Abstract] OR 'theta burst stimulation' [Title/Abstract] OR 'brain stimulation' [Title/Abstract] OR ' transcranial alternating current stimulation' [Title/Abstract]OR ' cerebellar stimulation ' [Title/Abstract]  #5 #1 AND #2 AND #3AND#4 | 35 |
| EMBase | #1 'cerebrovascular accident'/exp OR stroke OR apoplexy OR ‘brain infarction':ti,ab  #2 'locomotion'/exp OR 'lower limb'/exp OR gait OR 'lower extremit*' OR 'lower limb*' OR walking:ti,ab  #3  'robotics'/exp OR 'orthosis'/exp OR robot OR exoskeleton:ab,ti  #4  'nerve stimulation'/exp OR 'brain stimulation' OR ' cerebellar stimulation ' OR 'transcranial magnetic stimulation' OR ' transcranial alternating current stimulation ' OR ' cerebellar stimulation ' OR 'transcranial direct current stimulation':ti,ab  #5 #1 AND #2 AND #3AND#4 | 225 |
| CENTRAL (Cochrane Library) | #1 MeSH descriptor: [Stroke] explode all trees  #2 MeSH descriptor: [Gait Disorders, Neurologic] explode all trees  #3 (cerebrovascular disorder* OR stroke OR Cerebrovascular Accident* OR Apoplexy OR brain infarction*):ti,ab,kw  #4 MeSH descriptor: [Locomotion] explode all trees  #5 MeSH descriptor: [Lower Extremity] explode all trees  #6 (gait OR walking OR lower limb*):ti,ab,kw  #7 MeSH descriptor: [Robotics] explode all trees  #8 MeSH descriptor: [Exoskeleton Device] explode all trees  #9 MeSH descriptor: [Orthotic Devices] explode all trees  #10 (robot* OR orthotic* OR exoskeleton*):ti,ab,kw  #11 MeSH descriptor: [Transcranial Magnetic Stimulation*] explode all trees  #12 MeSH descriptor: [Transcranial Direct Current Stimulation*] explode all trees  #13 (nerve stimulation* OR ' cerebellar stimulation ' OR ' transcranial alternating current stimulation 'OR brain stimulation* OR TMS OR TDCS OR TACS):ti,ab,kw  #14 #1 OR #2 OR #3  #15 #4 OR #5 OR #6  #16 #7 OR #8 OR #9 OR #10  #17 #11 OR #12 OR #13  #18 #14 AND #15 AND #16 AND #17 | 113 |
| PedRO | stroke AND robot AND stimulation | 23 |
